# Surgery

**Published:** 1900-03

**Authors:** James Swain


### SURGERY.

**Internal derangement of the knee-joint,** or displaced semilunar cartilage—as the popularly accepted pathological phrase puts it,—is common enough; but according to Bennett[1] the symptoms in these cases are by no means always due to the cause which long familiarity has led us to adopt without questioning.

Of 200 cases presenting the characteristic symptoms—all cases which could be classified under the head of "loose bodies" being excluded, 112 were cured by temporary rest, massage and exercise; 83 without the use of any support or other apparatus; but in 39 temporary support was necessary for times varying from three months to one year. In no case in which the rest and massage treatment was commenced after the third attack was sufficient improvement effected to enable the patient, in the absence of operation, to entirely dispense with the use of support of some kind. The cases in which a support was worn permanently amounted to 58. Operation was performed in 27 cases: of these the semilunar cartilage was at fault in 15 only; of the remaining 12 the causes of the trouble were small pedunculated bodies in four instances, and abnormal folds of synovial membrane springing from or near the ligamentum mucosum in eight.

The symptoms usually recognised are: sudden pain, complete fixation or limitation of the power of extension of the leg upon the thigh, followed by effusion into the joint, with tenderness over one or other (nearly always the inner) semilunar cartilage. The only positive sign of the lesion being a displaced cartilage is the occurrence of a deficiency, upon comparing opposite limbs, felt above the head of the tibia on the affected side, in consequence of the displacement of the cartilage freely inwards. Relief may follow almost immediately upon spontaneous reduction of the displaced cartilage or nipped structure, or it may be effected by manipulation; or in some cases, principally the slighter ones, manipulation not only fails to give relief but may cause an increase of the symptoms.

The causes to which these symptoms are due are generally classified as follows: 1. Displacement of a semilunar cartilage. 2. The nipping of folds or shreds of synovial membranes between the ends of the bones. 3. Loose or pedunculated bodies in the joint. To these, however, Bennett adds a fourth, viz.:—Bruising of the peripheral edge of a semilunar cartilage and its

[1] *Lancet*, 1900, i. 6.

attachments without displacement or necessary loosening, the immediate result of the lesion being a local effusion of blood (subsequently reinforced by a certain amount of inflammatory exudation), a portion of which insinuating itself between the bone-ends acts as a foreign body. This is a new idea, and Bennett believes that it fully explains the train of symptoms in the majority of the milder cases of so-called "slipped cartilage." The reasons for this belief are as follows:—1. If the symptoms be due to a portion of semilunar cartilage, loose body, or fold of synovial membrane, they at once disappear, however pronounced they be, after reduction has been effected. In the majority of cases of the milder sort, however, in which the limitation of movement is only slight, "reduction of the displacement" cannot be effected by manipulation. Even under an anæsthetic it is impossible to completely straighten the limb, for it springs back into its former position directly the pressure is removed. The failure of attempts at "reduction" in these cases is due, not necessarily to any want of skill in the manipulation, but to the fact that there is nothing present which in the ordinary sense is reducible. 2. In spite of the apparent intractability of these cases at first, they invariably recover completely if properly treated, and do not recur. 3. The recovery of complete extension is *gradual*, never sudden as in the case when a foreign body has been withdrawn from between the bones. The gradual way in which the normal state is regained is in itself enough to negative the existence of anything like a foreign body in the ordinary sense of the term, between the bones, and the nature of the treatment by which recovery is brought about, added to the absence of any tendency to recurrence in properly managed cases, is strongly corroborative of this contention. 4. Cases submitted to operation have borne out the view here set forth, the semilunar cartilage being bruised and swollen but not detached.

Treatment may be conveniently considered under the following heads:—

1. *The Treatment by Temporary Rest, Massage and Exercise.*—In the exaggerated cases repeated attempts may be made, under chloroform if necessary, to effect "reduction"; but in the slight cases the attempt to restore complete extension should not be repeated if it fails on the first occasion. In all cases in which manipulation has effected reduction (severe cases), or has failed to reduce the deformity (slight cases), active movement should be prevented by complete temporary rest by fixation on a light splint for four days to a week—until the effusion in the joint has subsided. Massage of the joint may, however, be commenced at once; it hastens absorption, and obviates the tendency to flaccidity of the capsule of the joint, which is a fruitful cause of failure in these cases. Upon the disappearance of the effusion, exercise by passive movement is commenced, care being taken that no rotatory motion is imparted to the leg. The passive movement without rotation, whilst it does not disturb in any way the process of refixation, prevents any troublesome adhesion in the joint itself. Slight active movements are begun in about a fortnight, and at the end of three weeks from the date of injury the patient begins ordinary walking exercise. Massage must be kept up for three weeks to six months or more—until the capsule of the joint is restored to its normal condition of tension.

2. *Treatment by Apparatus.*—All forms of support or apparatus should be avoided, if possible, for they tend to render permanent the wasting of the thigh muscles. The cases in which the use of supports is justifiable are limited as follows:—(1) Cases in which, for unavoidable reasons, the rational treatment cannot be carried out; (2) cases in which the rational treatment fails to restore the normal tension of the knee capsule, or those in which abnormal lateral mobility remains to any marked extent in spite of treatment; and (3) cases in which recurrent attacks of semilunar displacement have occurred frequently,—operation having been for some reason rejected. The object to be obtained by artificial support is the prevention of rotation of the leg at the knee. Bennett thinks that the small spring "trusses" are useless; but I have found Paget's spring truss of much value in some of these cases.

3. *The Operative Treatment.*—In many cases it is practically impossible to determine the real cause of the trouble in intractable cases without an exploratory operation; but as many cases are curable without operation, the explorations should be reserved for (1) cases in which non-operative measures have failed; (2) cases in which general flaccidity of the joint, as shown by an abnormal amount of lateral movement on manipulation, is in excess of the other symptoms; (3) grossly neglected cases in which, from long-continued inflammation, the joint has assumed the aspect of pulpy disease; and (4) in certain cases of expediency (soldiers, etc.). Bennett thinks that nothing short of removal of the offending cartilage is efficient; but the operation is not without risk, and is not always perfect. An L-shaped incision is all that is necessary to give access to the part. Operation should not be undertaken while fluid is present in the joint. After operation massage of the limb above and below the knee is of importance in connection with flaccidity of the joint capsule, and passive movements of the knee may be commenced at the end of the first week.

**The surgical treatment of the ascites of cirrhosis** by the artificial production of peritoneal adhesions seems deserving of further trial, for comparatively few cases have been recorded since this method was introduced by Dr. Drummond and Mr. J. R. Morison.[1] Although it occasionally happens that cases

[1] *Brit. M J.*, 1896, ii. 728.

of ascites are apparently cured by repeated paracentesis, the majority do not so recover. It is probable, too, that many of the recoveries were not really cases of a non-inflammatory ascites such as occurs in cirrhosis of the liver, but were rather inflammatory effusions dependent upon some form of chronic peritonitis.

The method of operation consists in exposing the liver by an abdominal incision, and scraping the adjacent surfaces of the liver and diaphragm by means of the finger or a sponge. In some cases a ligature is passed through the edge of the liver, the omentum, and the cut parietal peritoneum, and the three structures are tied together so as to bring the omentum up between the liver and the diaphragm. This last procedure is regarded with favour by Dr. Rolleston and Mr. Turner,[1] because such stitching of the omentum, the liver, and the abdominal parietes together is more likely to be followed by effective vascular adhesions. In a brief account[2] of two cases they offer some remarks on the technique of the operation, and on the *rationale* of the treatment. Of these two cases, one of the patients improved, lost the ascites almost entirely, and felt much better in health, but was left with an enlarged spleen, so that he cannot as yet be regarded as cured. In the other case the ascites recurred again and again, and though the patient's general state is much better than before the operation, when it appeared that life would not be much prolonged, the result can at best be regarded as only slight amelioration.

It is important that the operation should be done comparatively early, and not postponed until the patient is too debilitated to withstand it, for patients with cirrhosis are prone to secondary infections, especially to peritonitis. Another reason for not delaying operative interference is the importance of intervening before the liver tissue is so degenerated that it is unable to undergo compensatory hyperplasia as the result of the improved blood-supply provided by the adhesions. Medical measures should only be persisted in while the diagnosis is open to doubt; and when a course of iodide of potassium has not benefited a case of ascites which is thought to be due to either syphilis or cirrhosis, medical treatment should be suspended and operative measures employed.

An oblique **incision** skirting the costal margin is recommended in preference to one in the semilunar line, because it appears to give better access to the upper surface of the liver on both sides of the suspensory ligament and to enable the omentum to be stitched on that surface between it and the diaphragm. **Suprapubic drainage** is considered necessary in cases where there is a large amount of fluid present and where there has been a rapid re-accumulation after previous tappings. In one of the cases reported by Dr. Rolleston and Mr. Turner, the enlarged spleen diminished in size after operation, and this they regard as an

[1] *Lancet*, 1899, ii. 1660. [2] *Ibid.*

indication for delay in adopting Talma's[1] practice of a second laparotomy and suture of the spleen to the abdominal parietes.

It has been assumed by Dr. Drummond and Mr. Morison,[2] and Dr. Weir,[3] that the general and local improvement depend simply on the collateral circulation relieving the pressure in the portal vein. Against this it may be argued (*a*) that ascites does not occur when the blood-pressure is presumably highest in the portal vein—viz., earlier in the course of the disease when hematemesis is met with; and (*b*) that ascites is a late manifestation and appears to be rather a result of a toxæmic condition of the blood than a mere mechanical result of increased portal blood-pressure, and that it is the outcome of a poison in the blood exerting a lymphagogue action. The toxæmic state depends on hepatic insufficiency—in other words, on the cirrhotic liver being unable to destroy poisons that are continually passing to it from the alimentary canal; and thus, though an increase in the collateral channels between the portal vein and the systemic veins might modify the engorgement of the portal vein, it would not tend to improve the general state of health, but would have rather the opposite effect. Inasmuch, therefore, as ascites is not a mere mechanical result of increased pressure in the portal vein, and as a very extensive communication between the portal vein and the general venous system leads to a deterioration of the general health, it appears that the good effects of the collateral circulation are not solely due to relieving the pressure n the portal vein.

It seems to Dr. Rolleston and Mr. Turner that there are two possible ways in which the development of a collateral circulation in adhesions around the liver may be beneficial to the economy. 1. By somewhat diminishing the flow of blood through the liver, it may enable that organ to deal more satisfactorily with the blood passing through it, and so reduce the toxæmic condition of the blood which is probably the important factor in inducing ascites. 2. That the increased vascular supply to the surface of the liver may, by improving the nutrition of the hepatic cells, enable them to undergo compensatory hyperplasia. The compensatory hypertrophy of the liver will enable the organ to perform more efficiently its important antitoxic functions and so lead to a latency of the symptoms. If the last hypothesis be true, it is evidently of importance that any operation for the formation of vascular adhesions around the liver should be undertaken before the liver tissue is so disorganised that compensatory hyperplasia is impossible.

In addition to the two successful cases here referred to, ten other cases have been published. Of these, at least three have died from the effects of the operation—two from peritonitis, and one from shock immediately after the cœliotomy.

JAMES SWAIN.

[1] *Berl. klin. Wchnschr.*, 1898, xxxv. 833. [2] *Loc. cit.*
[3] *Med. Rec.*, 1899, lv. 149.

